# A Flexible Neural Representation of Faces in the Human Brain

**DOI:** 10.1093/texcom/tgaa055

**Published:** 2020-08-28

**Authors:** Runnan Cao, Xin Li, Alexander Todorov, Shuo Wang

**Affiliations:** Department of Chemical and Biomedical Engineering, Rockefeller Neurosciences Institute, West Virginia University, Morgantown, WV 26506, USA; Lane Department of Computer Science and Electrical Engineering, West Virginia University, Morgantown, WV 26506, USA; Booth School of Business, University of Chicago, Chicago, IL 60637, USA; Department of Chemical and Biomedical Engineering, Rockefeller Neurosciences Institute, West Virginia University, Morgantown, WV 26506, USA

**Keywords:** dominance, face, face model, task modulation, trustworthiness

## Abstract

An important question in human face perception research is to understand whether the neural representation of faces is dynamically modulated by context. In particular, although there is a plethora of neuroimaging literature that has probed the neural representation of faces, few studies have investigated what low-level structural and textural facial features parametrically drive neural responses to faces and whether the representation of these features is modulated by the task. To answer these questions, we employed 2 task instructions when participants viewed the same faces. We first identified brain regions that parametrically encoded high-level social traits such as perceived facial trustworthiness and dominance, and we showed that these brain regions were modulated by task instructions. We then employed a data-driven computational face model with parametrically generated faces and identified brain regions that encoded low-level variation in the faces (shape and skin texture) that drove neural responses. We further analyzed the evolution of the neural feature vectors along the visual processing stream and visualized and explained these feature vectors. Together, our results showed a flexible neural representation of faces for both low-level features and high-level social traits in the human brain.

## Introduction

The face is one of the most important visual stimuli in the social world. In addition to gathering identity, gender, age, and emotion information from faces, people judge them on multiple trait dimensions (e.g., trustworthiness) effortlessly (Willis and Todorov 2006; Todorov et al. 2009). These judgements predict important social outcomes such as political elections (Todorov et al. 2005; Little et al. 2007; Ballew and Todorov 2007), criminal sentences (Blair et al. 2004; Eberhardt et al. 2006), and investment decisions (see Todorov et al. 2015 for a review). The functionality of face processing is supported by a dedicated neural system in primates (Haxby et al. 2000; Tsao et al. 2006), including regions of the occipital and posterior temporal visual cortices as well as subcortical structures such as the amygdala (Kanwisher et al. 1997; Jiang et al. 2006; Kanwisher and Yovel 2006; Kriegeskorte et al. 2007; Harris and Aguirre 2010; Natu et al. 2011; Axelrod and Yovel 2012; Mende-Siedlecki, Verosky et al. 2013; Avidan et al. 2014; Duchaine and Yovel 2015; Freiwald et al. 2016; Nestor et al. 2016; Grill-Spector et al. 2017; Rossion et al. 2018)

Although there is a plethora of literature on face perception, most of the existing studies focus on recognition of faces and emotional expressions. However, it remains largely unclear how the brain evaluates faces in general and how this evaluation is driven by low-level structural and textural features of the human face. Importantly, it remains unclear how explicit task demands or the evaluative context modulates such a general neural representation of faces. It has been shown that different types of face masks can modulate the amygdala’s response to facial emotions (Kim et al. 2010). Our own prior reports have not only found context-independent neural responses to facial trustworthiness (Wang et al. 2018) but also context-dependent (Sun, Zhen et al. 2017) and task-dependent (Sun, Yu et al. 2017) neural responses to facial ambiguity (see Todorov et al. 2011 for both task-dependent and task-invariant neural responses to facial trustworthiness). However, these prior studies only investigated a single face attribute and it remains to be investigated whether a general neural representation of faces is dynamically modulated by task instructions and evaluative goals.

To address this question, in the present study, in addition to traditional analysis of neural correlation with social traits, we employed a data-driven computational face model based on the face space approach (Leopold et al. 2001; Chang and Tsao 2017) that is able to identify and visualize the stimulus variation in faces that drives specific neural responses. This model is based purely on physical variation of the faces, without specifying a priori the importance of specific facial features (e.g., eye or mouth shape, skin texture, etc.), emotional content, or social meaning. In principle, any type of nonrandom brain response can be modeled as a function of this physical variation in the face stimuli. Importantly, our computational framework allows us to analyze and visualize representations at different levels of perception and cognition (e.g., functional magnetic resonance imaging [fMRI] versus behavioral data). Since these representations can be analyzed using the same metrics, their similarities and differences are immediately apparent. This allows us to simultaneously study the representations of faces in various brain areas at different levels of perception. Meanwhile, this also enables us to connect the findings from different blood-oxygen-level-dependent (BOLD) fMRI signals from this study to previously published literature. With this model, we not only surveyed the whole brain response that encoded significant facial feature vectors but also linked neural feature vectors with perceptual determinants of social attributes. In particular, we investigated how task instructions modulated neural representation of faces and identified brain areas that showed flexible neural representation given these different task instructions.

## Materials and Methods

### Participants

Thirty healthy volunteers (18 males, 18–31 years old, 3 left-handed) with normal or corrected-to-normal vision participated in this study. Two participants were excluded for further analysis due to excessive head motion (>6 mm) and another 3 participants were excluded because the localizer task failed to identify the fusiform face area (FFA). Written informed consent was given using procedures approved by the Internal Review Board of the West Virginia University (WVU).

### Stimuli

**Figure 1:**
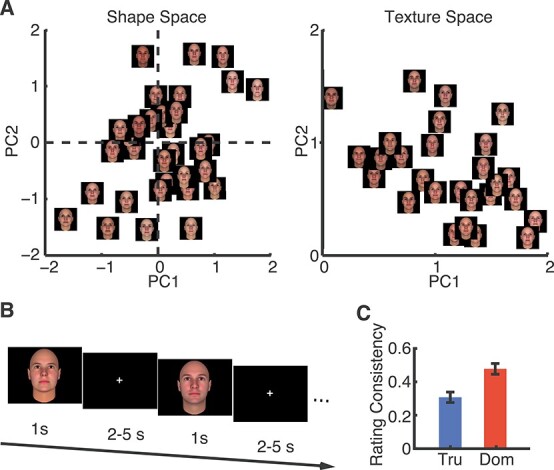
Stimuli, behavioral task, and consistency of judgments. (*A*) Computer generated face stimuli randomly varied on shape (e.g., round and oval) and skin texture (e.g., color) parameters: 50 shape and 50 texture PCs. Each PC is referred to as a feature of the face model, and it is also a dimension of the face space. Faces are thus represented as points in a 100-dimensional face space (50 shape and 50 texture dimensions). Moving a point along a single dimension changes the shape or texture of a face in specific ways. The average face is at the origin (center) of the face space. (*B*) Task; each face was presented for 1 s, followed by participants’ judgment of trustworthiness or dominance within 2 s. The overall inter-stimulus-interval was jittered between 2 to 5 s. (*C*) Correlation between the ratings from our fMRI participants with consensus ratings from (Oosterhof and Todorov 2008). (Pearson’s *r* = 0.33 ± 0.03 for trustworthiness and *r* = 0.50 ± 0.04 for dominance). Tru: trustworthiness. Dom: dominance.

In total, 300 Caucasian faces were randomly generated using the FaceGen Modeller program (http://facegen.com) version 3.1 (see Oosterhof and Todorov 2008 for detailed procedures). FaceGen constructs face space models using information extracted from 3D laser scans of real faces. To create the face space model, the shape of a face was represented by the vertex positions of a polygonal model of fixed mesh topology. With the vertex positions, a principal component analysis (PCA) was used to extract the components that accounted for most of the variance in face shape. Each principal component (PC) thus represented a different holistic nonlocalized set of changes in all vertex positions. The first 50 shape PCs were used to construct faces that had a symmetric shape (left panel of Fig. 1*A*). Similarly, because face texture is also important for face perception, 50 texture PCs (right panel of Fig. 1*A*) based on PCA of the red, green, and blue (RGB) values of the faces were also used to represent faces. The resulting 300 faces were randomly generated from the 50 shape and 50 skin texture components with the constraint that all faces were set to be Caucasian to avoid stereotypes in judgement. Note that only low-level features (face shape and skin texture) were manipulated to generate these face stimuli without referring to any higher-level social trait information.

Each PC is referred to as a feature of the face model, and it is also a dimension/axis of the face space. The feature axes of the spaces represent the features (e.g., shape and skin texture) with which faces are encoded. Each face has a value on each feature axis, and the set of values specify a face’s coordinates (position) in the face space. The reference of the face space is the prototype average face.

Notably, we have already acquired trait judgments of these faces from healthy control raters on 9 traits (Oosterhof and Todorov 2008) including trustworthiness and dominance. Therefore, these faces have benchmark ratings and we can readily perform correlational analysis with fMRI BOLD responses and psychometric behavioral data from the present study.

### Experimental Procedure

Stimuli were presented using MATLAB with the Psychtoolbox 3 (Brainard 1997) (http://psychtoolbox.org) (screen resolution: 1920 × 1080). Stimuli were presented onto an magnetic resonance imaging (MRI) compatible monitor and were viewed by participants via a mirror mounted atop the MRI head coil.

Each participant performed 2 face judgment tasks. In each task, there was a judgment instruction, that is, participants judged how trustworthy or how dominant a face was. We used a 1–4 scale: “1”: not trustworthy/dominant at all, “2”: somewhat trustworthy/dominant, “3”: trustworthy/dominant, and “4”: very trustworthy/dominant. We used a rapid event-related design (Fig. 1*B*). Each image subtending a 13^°^ visual angle was presented for 1 s at the center of the screen. Jittered interstimulus-interval ranging from 2 to 5 s was inserted after each image. Following each image, participants rated the level of dominance or trustworthiness of the face within 2 s. In total, 100 faces were presented in each run and there were 3 runs for each judgment task for a total of 6 runs. The order of stimuli was randomized and the order of tasks was counterbalanced across participants. At the beginning of each run, there was a fixation screen of 28 s to estimate the participant’s baseline BOLD signal. Task instructions were displayed at the beginning of each run.

Each participant performed a separate face localizer task after each judgement task to identify functional regions of interests (ROIs) that were selective to faces. We used a well-established task that displays video clips of dynamic faces, objects, and scrambled objects (see Pitcher et al. 2011 for details).

### Imaging Data Acquisition

MRI data were acquired on a 3 Tesla Siemens MRI Scanner (Prisma), with a 64-channel head coil, located at the WVU Rockefeller Neuroscience Institute. High-resolution T1-weighted anatomical images were collected using a 0.85 mm isotropic MPRAGE sequence (176 slices, centric phase encoding, acquisition matrix = 288 × 288, field of view = 250 × 250 mm, iPAT = 2, TR = 2040 ms, TE = 2.37 ms, TI = 1030 ms, flip angle = 8°). Functional data of the first 4 participants were acquired with the Siemens multiband echo planar imaging (EPI) sequence (TR = 2000 ms, TE = 30 ms, 2 mm isotropic voxels, field of view = 192 × 192 mm, image matrix = 96 × 96, iPAT = 2, flip angle = 80°, slice number = 52). Because this sequence had fewer slices and hence a smaller field of view, part of the superior frontal lobe could not be included in 3 of the first 4 participants. We, therefore, adjusted the sequence slightly (iPAT = 4, slice number = 64) for the rest of the participants to cover whole brain volume. Qualitatively the same results were derived if we excluded the first 4 participants for analysis. Padding cushions were provided around the head of participants to minimize head motion.

### Data Analysis: Behavior

For behavioral data, we calculated rating consistency for each individual by correlating his/her ratings with the average ratings from the previous study (Oosterhof and Todorov 2008), which served as the benchmark ratings (Fig. 1*C*). Since it has been reported that consensus ratings predict neural responses better than individual ratings (Engell et al. 2007), here we used the average ratings from (Oosterhof and Todorov 2008) for further analysis.

### Data Analysis: Imaging Data

MRI data were preprocessed and analyzed using Analysis of Functional NeuroImages (AFNI) (afni.nimh.nih.gov) and a custom MATLAB code. Anatomical T1 images were warped nonlinearly to the MNI template. The first 2 volumes of each functional run were discarded to allow the BOLD signal to reach a steady state. Preprocessing procedure involved despiking, slice timing correction, EPI distortion correction (PE blip-up), registration to the MNI template, rigid body motion correction, spatial smoothing (3 mm FWHM Gaussian Kernel), and scaling (as percent signal change).

We focused on the following ROIs as suggested by previous research (Todorov et al. 2011): the occipital face area (OFA), FFA, posterior superior temporal sulcus (pSTS), middle superior temporal sulcus, anterior superior temporal sulcus (aSTS), amygdala (Amyg), and inferior frontal gyrus (IFG). Face-selective areas were identified from the localizer task using the contrast of faces versus objects at the group level (Supplementary Fig. S1; *P* < 0.01 for all ROIs except *P* < 0.05 for the OFA, FFA, and IFG).

### Data Analysis: Modeling Social Traits

The neural representation of high-level social traits of trustworthiness and dominance was modeled separately using linear and quadratic regressors. The regressor for face onset (R1) and the regressor for consensus rating of each face (R2) were convolved with a hemodynamic response function to estimate 1) face evoked response, and 2) how the response to each face was modulated by perceived (consensus) trustworthiness or dominance (either linear or quadratic). Note that R2s were all demeaned to assure that R1 and R2 were not correlated. In addition, for all analyses, 6 head-motion regressors based on AFNI’s realignment estimation routine were included in the general linear model (GLM). The modulation of social attributes (i.e., trustworthiness and dominance) was estimated independently for each judgment task. For each task, we selected significant voxels from the task congruent conditions (e.g., voxels that were modulated by trustworthiness when the task instruction was to judge trustworthiness, and voxels that were modulated by dominance when the task instruction was to judge dominance) and predicted response for the task-incongruent social attributes (e.g., modulation by trustworthiness when the task instruction was to judge dominance).

Monte Carlo simulations using the function 3dClustSim in AFNI were conducted in order to estimate the null distribution of data and define the significant cluster size. Permutation with 10 000 runs was used to estimate the cluster size threshold for each ROI. We used a pervoxel threshold of *P* < 0.05 and a false positive rate (FPR) of null cluster lower than 0.05 as the cutoff for significance.

### Data Analysis: Constructing Neural Face Models

We used a simple linear model to identify the variation in faces that drove neural responses. The feasibility of this approach has been demonstrated in several prior studies (e.g., Oosterhof and Todorov 2008; Todorov et al. 2013) for modeling social judgments. Here, we used this same approach but changed the dependent measure using the BOLD signal from each voxel. Specifically, we fitted a GLM at face onset (regressor R1 above) and derived a beta value for each face. We averaged the beta values across participants to get a mean beta value for each face and each voxel. We then fitted a linear model for the mean beta values and calculated the vector of feature weights *w* as: *w* = *F* · *r,* where *r* is a column vector (*N* × 1) of the beta values to the *N* faces, and *F* is the feature matrix (each row is a feature and each column is a face) that contains the feature values for each face. We further normalized *w* by ||*w*||: *w* = *w*/||*w*||. The resulting feature vector *w* thus showed the optimal direction that best captured the variation in BOLD response.

Because a feature vector can always be derived given any response, we used linear regression to fit the neural response and determine whether a voxel had a significant feature vector. In addition, we used a permutation test with 1000 runs to further confirm significant voxels. For the data from each task, we randomly shuffled face labels and used 70% of faces as the training dataset. We used the training dataset to construct a model following the above procedure, predicted responses using this model for each face in the remaining 30% of faces (i.e., test dataset), and computed the Pearson correlation between the predicted and original response in the test dataset. The distribution of correlation coefficients computed with shuffling (i.e., null distribution) was compared to the one without shuffling (i.e., observed response). If the correlation coefficient of the observed response was greater than 95% of the correlation coefficients from the null distribution, this vector of feature weights was considered significant. Notably, both regression and permutation tests showed qualitatively the same selection of voxels. If a voxel encoded a significant feature vector, we subsequently derived feature weights as described above.

To control for multiple comparisons, we further employed a binomial test. Only when the number of significant voxels within an ROI was above the chance level (5% of the total number of voxels within an ROI), the cluster was retained for further analysis.

To present the feature tuning properties, we identified the feature index that had the largest feature weight in absolute value (i.e., the most preferred feature) in each significant voxel and ranked these voxels according to their preferred feature index. We also calculated a similarity representation map of feature weights across all significant voxels. Specifically, we correlated the feature weights of 1 voxel with all other voxels and organized voxels according to ROIs.

### Data Analysis: Validating And Explaining Neural Face Models

With the learned feature weights (*w*), we synthesized and visualized new test faces by reversing the feature extraction procedure. By construction, the specific changes in the synthesized faces could best capture the change in neural response. For example, the change in the face was reflected in linear changes (in standard deviation [SD] units) in both vertex positions that defined the face shape and colors (RGB) that defined the face texture.

One advantage of studying neural face models in humans is that we can further explain feature vectors using behavioral ratings. To explain the meaning of BOLD responses to physical variation in faces (e.g., whether the neural response encodes perceived trustworthiness of faces), we correlated the feature weights from the neural response with those from consensus judgments (Oosterhof and Todorov 2008). For example, if the neural feature weights were correlated with the feature weights derived from consensus trustworthiness ratings, it indicated that the neural response encoded trustworthiness. Furthermore, by separately correlating shape and texture weights, we could identify which physical change in faces drove the neural response. In addition, we correlated the feature weights from the neural response with those from our participants’ own ratings and derived qualitatively the same results (Supplementary Fig. S2).

### Data Analysis: Depth of Selectivity Index and Feature Selectivity

We quantified the depth of selectivity (DOS) for each voxel: $\mathrm{DOS}=\frac{n-\Big({\sum}_{j=1}^n{r}_j\Big)/{r}_{\mathrm{max}}}{n-1}$, where *n* is the number of features (*n* = 100), *r_j_* is the absolute feature weight to feature *j*, and *r*_max_ is the maximal absolute feature weight. DOS varies from 0 to 1, with 0 indicating an equal tuning to all features and 1 exclusive tuning to 1 feature, but not to any of the other features. Thus, a DOS value of 1 is equal to maximal sparseness of a feature weight.

## Results

### Task Modulation of Neural Encoding of Social Traits

We employed a social judgment task where participants rated the level of trustworthiness or dominance of each face during fMRI. The same 300 faces were rated in separate tasks for trustworthiness and dominance in counterbalanced order. Behaviorally, the ratings from our fMRI participants were consistent with the prior report (Oosterhof and Todorov 2008) for both trustworthiness and dominance (Fig. 1*C*). Therefore, we next used the consensus ratings as regressors to identify which face-selective brain areas encoded social traits of trustworthiness and dominance. We used both linear and quadratic regressors in the modeling according to previous studies (Todorov et al. 2008; Wang et al. 2018).

**Figure 2:**
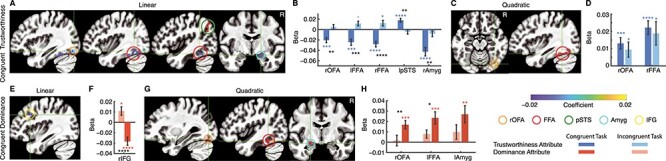
Task modulation of neural encoding of social traits. (*A, C*) Clusters within face-selective areas that significantly encoded the trustworthiness attribute linearly (*A*) and quadratically (*C*) when participants performed the trustworthiness judgment task. Overlaid is the mean coefficient of regression (cluster FPR < 0.05). (*B, D*) Mean parameter estimate (beta values) for the trustworthiness attribute across significant clusters. (*B*) Linear response. (*D*) Quadratic response. Blue asterisks indicate a significant difference from zero and black asterisks indicate a significant difference between congruent versus incongruent tasks. ^*^P < 0.05, ^*^^*^P < 0.01, ^*^^*^^*^P < 0.001, and ^*^^*^^*^^*^P < 0.0001. (*E, G*) Clusters within face-selective areas that significantly encoded the dominance attribute linearly (*E*) and quadratically (*G*) when participants performed the dominance judgment task. (*F, H*) Mean parameter estimate (beta values) for the dominance attribute across all significant voxels. (*F*) Linear response. (*H*) Quadratic response. Orange asterisks indicate a significant difference from zero and black asterisks indicate a significant difference between congruent versus incongruent tasks. ^*^P < 0.05, ^*^^*^P < 0.01, ^*^^*^^*^P < 0.001, and ^*^^*^^*^^*^P < 0.0001. Bars filled with dark colors refer to mean parameter estimates from the congruent task, and bars filled with light colors refer to mean parameter estimates from the incongruent task.

On the 1 hand, in the trustworthiness judgment task, we identified brain regions that encoded a linear change in facial trustworthiness (Fig. 2*A* and *B*; voxel-wise *P* < 0.05 corrected by cluster FPR < 0.05), including the bilateral FFA, left pSTS, and right OFA and amygdala (Table 1). Consistent with previous findings (Engell et al. 2007; Todorov et al. 2008), we found that the mean response of the right amygdala increased as a function of decreasing facial trustworthiness (Fig. 2*B*; *t*_(24)_ = 5.17, *P* < 0.0001). Furthermore, we identified additional brain regions in the face-selective areas that encoded a linear change of facial trustworthiness compared to previous studies (Engell et al. 2007; Todorov et al. 2008). Moreover, we also detected a quadratic response in the right OFA and FFA (Fig. 2*C* and *D* ; Table 1): the response was stronger to both trustworthy and untrustworthy faces than to faces in the middle of the trustworthiness dimension in these brain regions.

**Table 1:** Brain regions modulated by social traits

Social attribute	Response	ROI	Congruent task		Incongruent task	
			*t*_(24)_	*P*-value	*t*_*(*24)_	*P*-value
Trustworthiness	Linear	rOFA	−4.38	0.00020	1.00	0.32
		lFFA	−4.17	0.00034	−2.63	0.015
		rFFA	−5.12	<0.0001	−2.29	0.031
		lpSTS	−4.94	<0.0001	−0.96	0.34
		rAmyg	−5.17	<0.0001	−1.46	0.16
	Quadratic	rOFA	3.98	0.00055	2.16	0.041
		rFFA	5.56	<0.0001	2.79	0.010
Dominance	Linear	rIFG	−4.85	<0.0001	2.68	0.013
	Quadratic	rOFA	4.21	0.00031	0.31	0.76
		lFFA	3.96	0.00058	2.00	0.057
		lAmyg	3.24	0.0035	1.40	0.17

Note: All values from the congruent task (e.g., voxels modulated by trustworthiness during trustworthiness judgment) are *P* < 0.05 corrected by cluster FPR < 0.05.

On the other hand, none of the face-selective areas except a group of voxels in the right IFG encoded a linear change in facial dominance (Fig. 2*E* and *F*; voxel-wise *P* < 0.05 corrected by cluster FPR < 0.05; Table 1). However, the right OFA, left FFA, and left amygdala showed a quadratic response to dominance (Fig. 2*G* and *H*; Table 1), consistent with a previous study (Todorov et al. 2011).

Importantly, we next investigated whether neural encoding of social traits was modulated by context (e.g., the task at hand). In other words, we investigated whether neural response to facial trustworthiness or dominance was similar under different task instructions. To answer this question, we used the clusters identified from the congruent tasks (i.e., judgment instruction of the task was the same as the social trait; Fig. 2*A*, *C*, *E*, *G*) and tested whether these voxels still encoded the social trait in the incongruent task (i.e., judgment instruction of the task was different from the social trait). Note that the response predicted in the incongruent task was completely independent of the selection of voxels.

First, we found that none of the brain regions showing a significant linear response to facial trustworthiness in the trustworthiness judgment task responded similarly in the dominance judgment task (Fig. 2*B*; Table 1; 2-tailed paired *t*-test between congruent versus incongruent tasks; right OFA: *t*_(24)_ = −3.57, *P* = 0.0016; left FFA: *t*_*(*24)_ = −4.39, *P* = 0.0002; right FFA: *t*_(24)_ = −4.85, *P* < 0.0001; left pSTS: *t*_(24)_ = 3.48, *P* = 0.002; right amygdala: *t*_(24)_ = 3.00, *P* = 0.007), suggesting that these brain regions encoded facial trustworthiness in a flexible manner and their response was modulated by task instructions. Note that the bilateral FFA even responded in the opposite direction in the incongruent task. Interestingly, both right OFA and FFA still showed a significant quadratic response in the incongruent task as in the congruent task (Fig. 2*D*; Table 1; right OFA: *t*_(24)_ = 0.70, *P* = 0.50; rFFA: *t*_(24)_ = 0.63, *P* = 0.54).

Second, the only brain region that showed a significant linear response to facial dominance in the dominance judgment task (i.e., the right IFG) responded in the opposite direction (*t*_(24)_ = 2.68, *P* = 0.013) in the trustworthiness judgment task (Fig. 2*F*; *t*_(24)_ = 5.71, *P* < 0.0001). Furthermore, we found that none of the brain regions showing a significant quadratic response to facial dominance in the dominance judgment task still encoded such quadratic response any more in the incongruent task (Fig. 2*H*; right OFA: *t*_(24)_ = 3.31, *P* = 0.003; left FFA: *t*_(24)_ = 2.78, *P* = 0.01; left amygdala: *t*_(24)_ = 1.83, *P* = 0.08), again suggesting a flexible encoding of social traits.

Taken together, these data suggest the identification of brain regions in the face-selective areas that encoded facial trustworthiness and dominance while primarily encoding social traits in a flexible manner.

### Task Modulation of Neural Representation of Low-Level Face Features

In addition to task modulation of neural encoding of social traits, do different tasks also lead to different neural representation of low-level face features? To answer this question, we employed a face modeling approach (see Methods), which enabled us to readily study what variations in stimulus drive neural responses to faces. This approach is completely data-driven and is able to reveal the physical changes in the face (i.e., low-level features of face shape and skin texture) that can best explain the neural response. Because the construction of neural face models does not require any a priori assumptions about regressors or behavioral responses from the participants, it can derive a more general neural representation of faces compared to the above regression analysis with social traits. Therefore, the face modeling approach can extend our knowledge of face representation in the brain by revealing a generic neural code of face processing.

**Figure 3:**
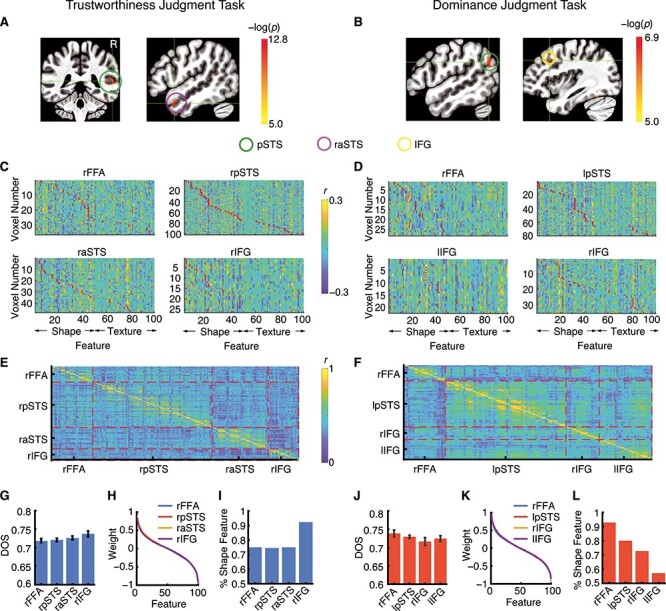
Face modeling. (*A, B*) Brain regions within face-selective areas that encoded significant feature vectors. We performed a linear regression between neural response (i.e., beta values) and face features to determine whether a voxel encoded a significant feature vector and further controlled for multiple comparisons using a binomial test. We then calculated the feature weights using our face model (see Methods). Overlaid is the log transformation of *P*-values. (*C, D*) Feature weights for all significant voxels in the clusters that survived the binomial test. Color coding shows the feature weight values. Red dots indicate the most preferred feature of that voxel (i.e., the feature with the largest weight in absolute value). Voxels are ordered according to the most preferred feature. Features 1–50 are shape features. Features 51–100 are texture features. (*E, F*) Representation similarity matrix for each pair of significant voxels. Significant voxels were arranged by face-selective areas (shown by red dashed lines). (*G, J*) Summary of DOS. Error bars denote 1 SEM across voxels. (*H, K*) Ordered average feature weights for each cluster. Feature weights were normalized by the largest absolute feature weight. (*I, L*) Percentage of voxels that most preferred a shape feature. (*A, C, E, G, H, I*) Trustworthiness judgment task. (*B, D, F, J, K, L*) Dominance judgment task.

For each judgment task, we observed clusters of voxels within face-selective areas that encoded significant feature vectors (see Methods for cluster selection). These clusters were distributed along the visual stream, including the right FFA, bilateral pSTS, right aSTS, and bilateral IFG (Fig. 3*A* and *B*). Clusters identified from different judgment tasks were largely separate, with less than 10% overlapping voxels for each cluster, again suggesting a flexible neural face representation that varied as a function of task instruction. We next investigated the pattern of features in these clusters.

First, we found a general consistency of feature patterns across voxels within each cluster (see “stripes” in Fig. 3*C* and *D*; color-coding indicates feature weight values), although the most preferred feature (i.e., the feature with the largest weight; shown by red dots in Fig. 3*C* and *D*) varied across voxels. Specifically, the right FFA encoded various shape and texture features (note the difference between task instructions; see below), consistent with the previous literature showing that the FFA (especially the right FFA) is sensitive to shape and texture features (Jiang et al. 2009; Harris et al. 2014) (note that in line with our results, the right FFA shows a stronger response to shape features, Jiang et al. 2009). In contrast to our findings that the pSTS is sensitive to both shape and texture features, a previous report has shown that the pSTS is only sensitive to shape but not texture features (Harris et al. 2014). However, using emotional faces, it has been shown that both shape and texture properties predict response to facial expressions in the pSTS (Sormaz et al. 2016), consistent with our present findings. Furthermore, we found that the IFG encoded various shape and texture features (note the difference between task instructions; see below), in line with the previous literature showing that the IFG is sensitive to both shape and texture features (Jiang et al. 2009).

Second, for both judgment tasks, voxels from the same cluster showed a more similar feature pattern (shown by a higher correlation of feature weights; Fig. 3*E* and *F*) whereas voxels from different clusters showed less similar feature patterns (Fig. 3*E* and *F*).

Third, feature selectivity (i.e., distribution of feature weights) was similar across clusters, as shown by the similar DOS index (Rainer et al. 1998) (see Methods; Fig. 3*G*, *J*; 1-way ANOVA across clusters: *F*_(3,208)_ = 1.36, *P* = 0.26 for trustworthiness and *F*_(3,161)_ = 1.18, *P* = 0.32 for dominance; note that DOS values range between 0 and 1, with 1 indicating tuning to only a single feature and 0 indicating an equal tuning to all features), as well as similar ordered feature weights from the most preferred to the least preferred features for these voxels (Fig. 3*H*, *K*; note that the steepness of change in these ranked curves indicates the level of selectivity).

Lastly, we investigated whether shape features (features 1–50) and texture features (features 51–100) were preferably encoded in different brain regions as shown in previous studies (Chang and Tsao 2017). We calculated the percentage of significant voxels that had a shape feature as the most preferred feature (shown by red dots in Fig. 3*C* and *D*). We found that in the trustworthiness judgment task, shape features were more preferred in the right IFG (Fig. 3*I*), whereas in the dominance judgment task, there was a decreasing trend of preference for shape features along the visual processing stream (Fig. 3*L*; see also Fig. 3*C* and *D* for examples). Our results again suggested that there was a difference in the representation of face information for different tasks.

Together, we identified brain regions that encoded significant feature vectors, that is, voxels whose response followed purely the physical change in stimulus rather than its psychological meaning, and we also revealed the evolution and properties of neural face representation along the visual stream. We next explored the meaning of such face representation.

### Visualizing and Explaining Visual Face Models

**Figure 4:**
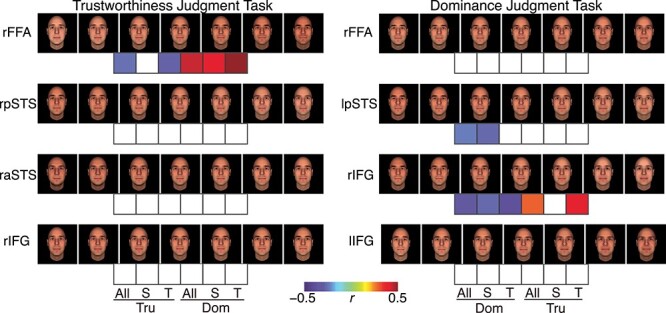
Visualization and explanation of feature vectors. Shown are synthesized faces (ranging from −9 SD to +9 SD) using average feature weights from all voxels that encoded significant feature vectors. Left: trustworthiness judgment task. Right: dominance judgment task. Color bars below faces indicate correlation strength (i.e., correlation coefficient) between feature weights derived from neural responses (the ones that are used to synthesize faces) and feature weights derived from consensus social trait ratings. Only significant correlations are shown with colors. All: correlation using all features; S: correlation using shape features only; T: correlation using texture features only; Tru: trustworthiness ratings; Dom: dominance ratings.

Our face model allows us to readily visualize what physical changes in the stimulus best drive the neural response and easily compare feature vectors derived from different response modalities (e.g., comparing neural feature vector with behavioral judgment feature vector). Here, we capitalized on these advantages by synthesizing new faces that illustrated what changes in faces best drove the neural response (Fig. 4). We used the mean feature vector (i.e., averaging feature weights across voxels) from all significant voxels from a cluster because the significant voxels from a cluster showed a general consistency (Fig. 3*C*–*F*).

Although different brain regions are widely considered to be involved in face processing, we found that these regions encoded different changes in faces (Fig. 4), suggesting that different brain regions process different properties of faces. The synthesized faces showed changes in shape (i.e., configuration of different facial parts) as well as in texture (the color of the face). In particular, we found the following trends: 1) the dominance judgment task (Fig. 4 right) tended to elicit a greater change in faces in general compared to the trustworthiness judgment task (Fig. 4 left). 2) Some brain regions were modulated by both shape and texture features (e.g., the rFFA in the trustworthiness judgment task) whereas other brain regions were primarily modulated by only shape or texture feature (e.g., the rIFG in the trustworthiness judgment task was primarily modulated by the shape feature). Notably, consistent with our findings, the FFA has been shown to be sensitive to both shape (Jiang et al. 2009) and texture (Jiang et al. 2009; Harris et al. 2014) features and the pSTS has been shown to encode both shape and texture features (Sormaz et al. 2016) (see our results from pSTS in both tasks; Fig. 4). 3) Changes in faces tended to be more subtle (i.e., smaller variation in faces) along the visual processing stream in the trustworthiness judgment task. More importantly, by comparing the synthesized faces from the same brain region under different task instructions (e.g., the rFFA as well as the rIFG), we found that the brain region represented vastly different faces, suggesting a flexible encoding of faces under different task instructions.

We next correlated neural feature vectors with social trait behavioral judgment feature vectors (derived using consensus ratings from Oosterhof and Todorov 2008) to explore whether the physical changes in faces could be explained by any social perceptions. We found several correlations (Fig. 4). In the trustworthiness judgment task, the neural feature vector from the right FFA was correlated with behavioral judgment feature vectors of trustworthiness and dominance, suggesting that this neural feature vector contained information about trustworthiness and dominance judgments. In the dominance judgment task, the neural feature vector from the left pSTS was correlated with behavioral judgment feature vectors of dominance while the neural feature vector from the right IFG was correlated with behavioral judgment feature vectors of trustworthiness and dominance. Note that the behavioral judgment feature vectors of trustworthiness and dominance have previously been shown to be negatively correlated with each other (Oosterhof and Todorov 2008), therefore it is not surprising to observe a neural feature vector correlating with both behavioral judgment feature vectors simultaneously. Furthermore, we derived similar results using judgments of faces from participants in the present study (Supplementary Fig. S2). We found additional correlations in the left pSTS and right IFG with trustworthiness. Lastly, it is worth noting that our face modeling revealed physical changes in faces that significantly modulated neural responses, though such physical changes might not be directly associated with a specific social percept (e.g., explained by a social trait). We therefore did not expect to find significant correlations with behavioral judgment feature vectors for all neural feature vectors.

Together, we observed the physical changes in faces that drove the neural response. Compared to previous neuroimaging literature (Jiang et al. 2009; Harris et al. 2014; Sormaz et al. 2016), we directly modeled the physical changes in faces without resorting to the fMRI adaptation procedure and thus our present results provided a more comprehensive analysis of face features and revealed more subtle changes in faces that could drive neural responses in specific ROIs. We also found that a subset of neural feature vectors also contained information about social traits such as trustworthiness and dominance.

### Summary of Results

In summary, we found that neural representation of faces for both social traits and low-level facial features showed a vastly different pattern based on different task instructions, suggesting a flexible and dynamic encoding of faces. Specifically, our results can be summarized as follows:

1. Different face-responsive brain regions encoded social traits and low-level features of face differently: 1) the FFA (especially the rFFA) encoded both social traits (trustworthiness and dominance) and low-level features (shape and skin texture) and in both cases the FFA showed a different response in different tasks. 2) The STS (especially the pSTS) primarily encoded low-level features, but the right pSTS was involved in the trustworthiness judgment task whereas the left pSTS was involved in the dominance judgment task. 3) The amygdala was primarily involved in encoding social traits but not low-level features. 4) The IFG (especially the right IFG) primarily encoded low-level features (although it was the only region that encoded the quadratic response of the dominance trait; Fig. 2*E*, *F*) where it showed a different response in different tasks and the left IFG only encoded low-level features in the dominance judgment task.
2. The brain regions encoding low-level features showed a different pattern of feature selectivity (Fig. 3*G*, *J*) and feature preference (Fig. 3*I*, *L*) for different tasks. Only in the dominance judgment task, there was a decreasing trend of shape preference along the visual processing stream (but the trustworthiness judgment indicated an opposite trend). Interestingly, we found that primarily the right hemisphere encoded low-level features in the trustworthiness judgment task (Fig. 3*C*, *E*) whereas both hemispheres were involved in the dominance judgment task (Fig. 3*D*, *F*). Note that the right FFA and right IFG encoded low-level features in both tasks, although different voxels were involved.
3. The encoded changes in the synthesized faces were diverse across different brain regions, and the changes seemed to become more subtle along the visual processing stream. Only the low-level features encoded by the right FFA in the trustworthiness judgment task and the left pSTS and right IFG in the dominance judgment task could be explained by behavioral judgment of social traits.

## Discussion

In this study, we used fMRI to identify brain regions that encoded high-level social traits such as facial trustworthiness and facial dominance and that these brain regions were modulated by task instructions. We then employed a face modeling approach and identified brain regions that encoded low-level variation in structural and textural features of the faces that drove neural responses. We further analyzed the evolution of the neural feature vectors along the visual processing stream and visualized and explained these feature vectors. Together, our results showed a flexible neural representation of faces for both low-level facial features and high-level social traits in the human brain.

### Possible Caveats

Consistent with previous functional neuroimaging findings (Todorov et al. 2011) (see Mende-Siedlecki, Said et al. 2013 for a meta-analysis), in the trustworthiness judgment task, we observed a linear effect of trustworthiness in the right OFA, bilateral FFA, right amygdala, and left pSTS, and a quadratic effect in the right OFA and FFA. However, in the dominance judgment task, we observed a linear effect of dominance in the right IFG, which has only been shown to change activity according to trustworthiness but not dominance in previous studies (Mende-Siedlecki, Said et al. 2013, Todorov et al. 2011). Furthermore, consistent with our present results, previous studies have shown a quadratic effect of dominance in the left FFA (Mende-Siedlecki, Said et al. 2013, Todorov et al. 2011); but we also observed a quadratic effect of dominance in the right OFA and left amygdala in the present study. The difference with the previous literature was likely due to the different tasks used (also see below), the major conclusion of our present study. It is also worth noting that the encoding of social traits and low-level features could be differently sensitive to task instructions (see summary of results) and the beta coefficients could be even reversed in incongruent tasks (Fig. 2). Again, both of these may have resulted from the highly flexible encoding of faces under different task demands and contexts.

When we synthesized faces for visualization and explanation, we averaged the model parameters (i.e., feature weights) across all voxels that encoded a significant feature vector, given the consistency in model parameters across voxels (Fig. 3). Therefore, these feature vectors represented the mean response or the most dominant response within a cluster. However, each significant voxel may encode different information of faces, and even single cells within a brain region can encode different information (Chang and Tsao 2017). Therefore, a future study with multivariate analysis will be needed to investigate how these feature vectors that are encoded by different units collectively lead to face perception.

In this study, we used a computer-generated model faces in order to parametrically vary the faces in the face space (Fig. 1*A*). Rapid advances in computer vision and the development of deep neural networks (DNNs) have provided an unprecedented opportunity to extract features from real human faces and subsequently manipulate these features to generate new photorealistic faces while providing well-controlled stimuli to investigate differences in neural responses to feature changes (see O'Toole et al. 2018 for a review). Therefore, a clear future direction is to apply our face modeling approach with real human faces with face features extracted by DNNs.

### Advantages of Our Approach

Our face modeling approach allows the study of facial features for face identity, emotional expressions, and social traits. Importantly, it allows us to study task modulation of such neural representations. Notably, our computational face models have the following advantages: 1) they are free of any prior assumption about what feature or content is important to trigger neural responses and they can model any nonrandom response to faces. This is also a general approach, which is able to not only analyze low-level features but also high-level features such as social traits by correlating with the feature vectors derived using the corresponding social judgment. Therefore, responses to social traits can also be analyzed under this framework. 2) They are independent of explicit judgments, mimicking the real-world scenario where people form instantaneous impressions of others. 3) They are able to integrate responses from different measurements, such as behavioral judgments, eye movements, and fMRI BOLD responses. Because all measures can be transformed to the same metric (feature weights), we can directly analyze the similarity and link the computations between measurements. 4) They can analyze the temporal dynamics of face representation. 5) They can synthesize new faces for testing on a variety of dimensions. 6) Rather than elementalizing features/changes in faces (e.g., Freiwald et al. 2009), they analyze holistic changes in faces (e.g., Chang and Tsao 2017; VanRullen and Reddy 2019). Together, our computational face model is capable of not only revealing the physical variation in the face that determines complex face evaluations but is also capable of identifying the neural basis of these face evaluations. Such computational modeling approach has been shown to be very useful for reconstructing faces from neural signals (Lee and Kuhl 2016; Nestor et al. 2016; VanRullen and Reddy 2019). Furthermore, our computational framework and data-driven methods can be easily extended to characterize any set of complex 3D objects that can be generated by a low-dimensional manifold parameterized in high-dimensional space (e.g., Kurosu and Todorov 2017).

### Flexible Versus Invariant Coding of Faces

In this study, we found primarily flexible neural representation of faces for both low-level and high-level face features. Our present findings are consistent with our previous reports that the neural signature indexing facial ambiguity is modulated by context (Sun et al. 2017b) and task instructions (Sun et al. 2017a). Our results are also in line with the finding that the amygdala processes stimulus relevance and evaluative goals and that it can be dynamically modulated by motivations of the perceiver (Cunningham et al. 2008; Cunningham and Brosch 2012). Furthermore, the IFG shows flexible neural coding during categorical decision making by shaping its selectivity to reflect the behaviorally relevant features (Li et al. 2007). On the other hand, in the present study, we found that the right amygdala showed flexible response to facial trustworthiness given different task instructions, in contrast to our prior report that the right amygdala’s response to trustworthiness is not modulated by stimulus range or social context (Wang et al. 2018). This is likely because here we used judgment of trustworthiness versus dominance, 2 largely orthogonal social traits (Oosterhof and Todorov 2008), whereas we previously used “approach” versus “avoidance” task instructions (Wang et al. 2018), which were both based on facial trustworthiness. In addition, consistent with the present results, a prior report using approach-avoidance versus 1-back recognition tasks has identified brain regions that are invariant or flexible to evaluation of social traits (Todorov et al. 2011). More broadly, top-down factors such as task instructions, task demands, and evaluative context have been shown to impact face processing even in an implicit manner (Ratner et al. 2012; Collins et al. 2016; Stolier and Freeman 2016). A future study using multi-voxel pattern analysis (MVPA) may reveal more subtle information about how faces are neurally represented in different contexts (Ratner et al. 2012; Collins et al. 2016; Stolier and Freeman 2016).

### Face Space and Feature-Based Coding

There have been efforts to identify responses to axes of biologically or computationally derived “face space” models, in which each face is represented as a vector in a multi-dimensional space. Along these lines, we have shown that the human brain can represent certain dimensions of facial expressions such as intensity (Todorov et al. 2008; Wang et al. 2017). Notably, there has been a long history of using a face space approach to test feature-based models and study face representations in the brain in general. This approach has been demonstrated as a useful tool in many studies investigating face perception. For example, using the face space approach, it has been shown that adaptation specifically shifts the perception along a trajectory passing through the adapting and averaging faces, selectively facilitating recognition of a test face lying on this trajectory and impairing recognition of other faces (Leopold et al. 2001). Such norm-based face encoding is supported by neuronal evidence from the monkey inferotemporal cortex (Leopold et al. 2006) and human fMRI (Loffler et al. 2005): individual faces are encoded by their direction (facial identity) and distance (distinctiveness) from a prototypical (average) face, and when facial geometry (head shape, hairline, internal feature size, and placement) is varied, the fMRI signal gets larger with increasing distance from the average face (Loffler et al. 2005). In addition, there is a face feature space in the macaque temporal lobe (Freiwald et al. 2009), and a recent study has shown that each face cell’s firing rate is proportional to the projection of an incoming face stimulus onto a single axis in the face space, allowing a face cell ensemble to encode the location of any face in the space (Chang and Tsao 2017).

In conclusion, our present results of face modeling (Figs. 3 and 4) support feature-based coding, where physical changes in faces parametrically modulated neural responses. Furthermore, we found that various brain regions within the face-selective areas were parametrically modulated by social traits (Fig. 2), again supporting feature-based coding. Together, we identified brain regions that parametrically encoded both low-level structural and textural features of faces as well as high-level semantic meaning of social traits, and we further found that such feature-based coding was flexible given different task instructions. Future studies will be needed to compare these competing face coding schemes at the single-neuron or neural population level.

## Notes

We thank Paula Webster for valuable comments. *Conflict of Interest*: The authors declare no conflict of interest.

## Funding

This research was supported by an NSF CAREER Award (1945230), ORAU Ralph E. Powe Junior Faculty Enhancement Award, West Virginia University (WVU), WVU PSCoR Program, and the Dana Foundation (to S.W.), and an NSF Grant (OAC-1839909) and the WV Higher Education Policy Commission Grant (HEPC.dsr.18.5) (to X.L.). The funders had no role in study design, data collection and analysis, decision to publish, or preparation of the manuscript.

## Author Contributions

R.C., A.T., and S.W. designed research. R.C. performed research. R.C. and X.L. analyzed data. R.C., A.T., and S.W. wrote the paper. All authors discussed the results and contributed toward the manuscript.

## Supplementary Material

Supplementary_Materials_tgaa055
