# Supplementary material for: A Flexible Neural Representation of Faces in the Human Brain
Source: Cereb Cortex Commun. 2020 Aug 28;1(1):tgaa055. doi: 10.1093/texcom/tgaa055 (PMC8152845; doi:10.1093/texcom/tgaa055)
Supplement: Supplementary_Materials_tgaa055 [file supplementary_materials_tgaa055.pdf]

Supplementary Figures

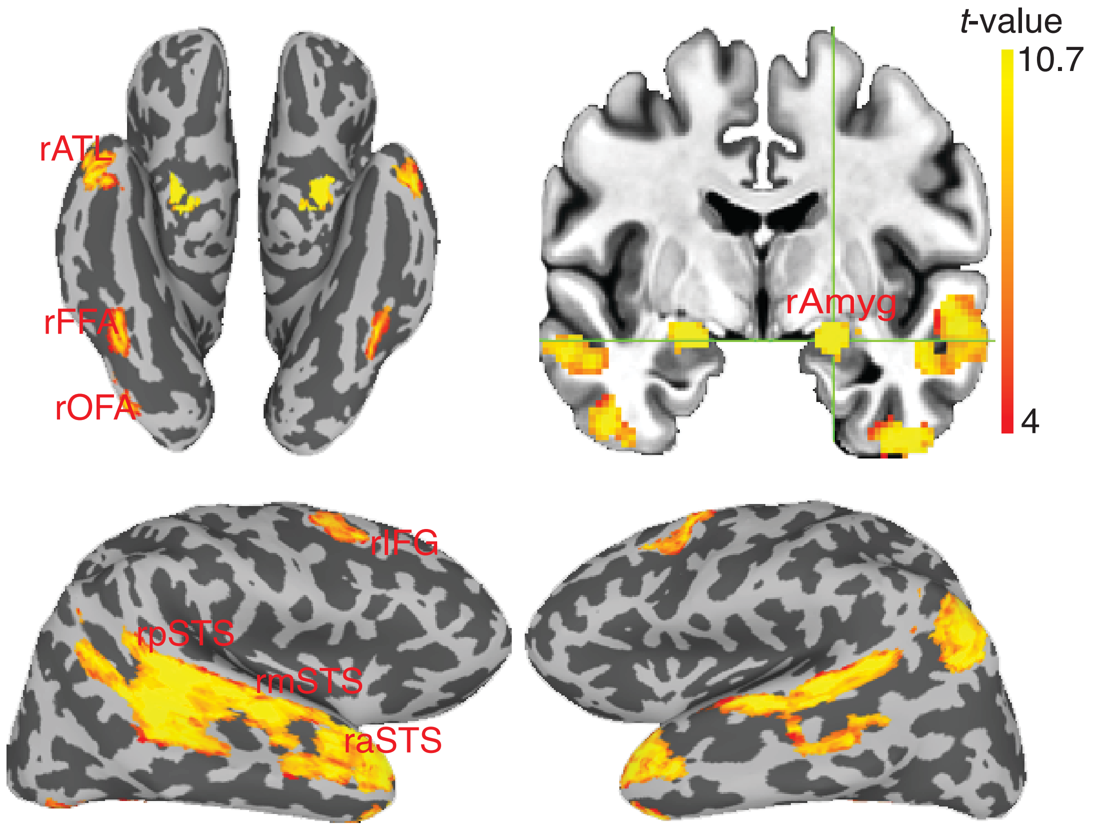

Figure S1. Face-selective areas identified using the face localizer task. The face−object condition revealed significant differences in brain areas including the occipital face area (OFA), fusiform face area (FFA), posterior, middle, and anterior superior temporal sulcus (STS), amygdala, and inferior frontal gyrus (IFG). Shown are group results of all participants.

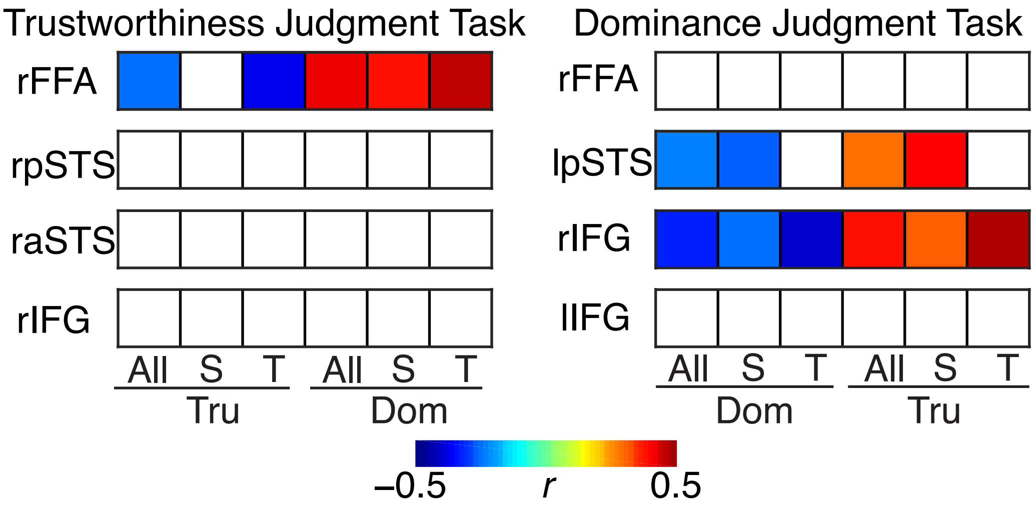

Figure S2. Correlation between feature weights derived from neural response with feature weights derived from our own participants' ratings. Legend conventions as **Fig. 4**.
